# On strongly connected networks with excitable-refractory dynamics and delayed coupling

**DOI:** 10.1098/rsos.160912

**Published:** 2017-04-05

**Authors:** P. Grindrod, T. E. Lee

**Affiliations:** Mathematical Institute, University of Oxford, Oxford OX2 6GG, UK

**Keywords:** invariant attracting tori, computational simulation, resonance, phase locking, decision-making

## Abstract

We consider a directed graph model for the human brain’s neural architecture that is based on small scale, directed, strongly connected sub-graphs (SCGs) of neurons, that are connected together by a sparser mesoscopic network. We assume transmission delays within neuron-to-neuron stimulation, and that individual neurons have an excitable-refractory dynamic, with single firing ‘spikes’ occurring on a much faster time scale than that of the transmission delays. We demonstrate numerically that the SCGs typically have attractors that are equivalent to continual winding maps over relatively low-dimensional tori, thus representing a limit on the range of distinct behaviour. For a discrete formulation, we conduct a large-scale survey of SCGs of varying size, but with the same local structure. We demonstrate that there may be benefits (increased processing capacity and efficiency) in brains having evolved to have a larger number of small irreducible sub-graphs, rather than few, large irreducible sub-graphs. The network of SCGs could be thought of as an architecture that has evolved to create decisions in the light of partial or early incoming information. Hence the applicability of the proposed paradigm to underpinning human cognition.

## 1. Introduction

There has been an explosion in the number of mathematical and computer models proposed to describe a wide range of neurological phenomena [1]. However, the nervous system is composed of approximately 10^11^ neurons [2], a network far too large to model directly. To overcome this, and to make use of high- (though limited) resolution data, some models have considered smaller graphs, where each node represents a region of the brain. For example, McIntosh *et al.* [3] use as few as four nodes and Grindrod *et al.* [4] use 10^5^, where each node is the voxel from an fMRI scan. Other observational methods use multivariate time series, such as readings from EEGs, MEGs and fMRIs [5]. These readings are essentially representing the brain as communication between approximately 64 regions. Region-to-region communication is tracked by measuring activation level, firing rate or membrane potential across time. However, the signals are not on the neuron level, they are noisy and involve conduction delays. Methods to analyse these time series include phase lag index, phase-locking value and partial directed coherence [6–8]. Complex networks of multivariate time series have undergone a rapid growth in recent years, and have been successfully applied to solve challenging problems in many research fields, and brain network is one of them. In particular, Gao *et al.* [9] proposed a multiscale complex network, a multi-frequency complex network [10], a multiscale limited penetrable horizontal visibility graph [11] (an alternative to the visibility graph [12]) and a multivariate weighted complex network [13] to analyse multivariate time series. These methods have proved to be powerful analytic frameworks for characterizing complicated dynamic behaviours from observable time series. Nonetheless, these observational approaches are essentially providing a top-down analysis. That is, they inform us where energy is being used, but provide no details about what processes are actually taking place.

So although analysing structural networks may help us to understand the fundamental architecture of inter-regional connections, we must also consider functional networks directly to elucidate how this architecture supports neurophysiological dynamics [14]. Inspired by the advent of micro-electrode recording techniques, which made it possible to record electrical events from single neurons, many models focus on systems composed of two or three neurons ([1] and references therein). Let us elaborate on models of this type.

**Figure 1.**
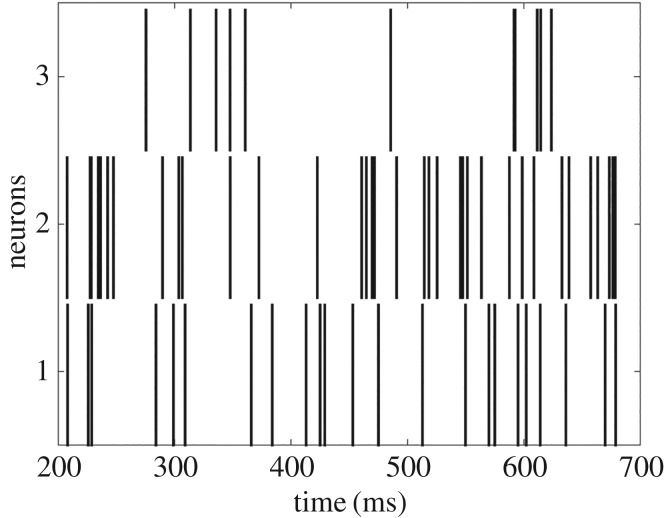
Anexample of three neural spike trains.

For models where only two to three nodes are considered, a common starting point is the relationship between the neuron’s input and output. The times of neuron firings can be presented as a neural spike train (figure 1). Considerable experimental and theoretical work has been devoted to measuring and modelling the statistical properties of neural spike trains in the hope of deciphering the neural code [15,16]. Commonly, the frequency or inter-spike interval is analysed; for example, inter-spike intervals for many neurons can be described by density functions, which may be generated by chaotic deterministic processes [17]. There is experimental evidence to suggest that even very random looking neural spike trains may represent deterministic chaos [18].

Recent developments in the quantitative analysis of complex networks, based largely on graph theory, have been rapidly translated to studies of brain network organization [14]. Graph theory offers new ways to quantitatively characterize anatomical patterns. Structural neural networks can be described as directed graphs that are composed of nodes denoting neurons that are linked by edges representing physical connections (synapses or axonal projections) [14]. The notion that structure can predict function also informs one of the chief goals of connectomics, which is to furnish network models that can bridge brain structure and function [19,20].

**Figure 2.**
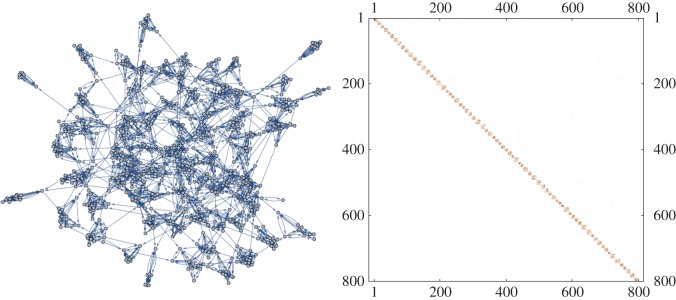
A directed network containing many SCGs together with its block upper triangular adjacency matrix. We model the neural network by examining the activity within the SCGs, where we experiment with varying sizes of SCGs.

Our work is in the spirit of the theoretical models of two or three nodes, but on a considerably larger scale. We consider the neuronal network on the micro-scale (neurons), the meso-scale (strongly connected subgraphs, SCGs) and the macro-scale (SCGs connected loosely as a whole, figure 2). Our contribution is to quantify how behaviour on the micro-scale impacts the meso-scale. We simulate the micro- and meso-scale behaviour by considering the individual excitable and refractory neuron firing times, via neural firing trains, but on a scale much larger than two or three nodes. By computationally simulating firing times, we record many neural firing trains and represent this time series in an *n*-dimensional space. We find that all else being equal, the functionality of SCGs increases sublinearly with the size of the SCG. This has profound implications with regard to explaining how brains evolved to ensure high functionality.

The structure of our network is further described in §2. The dynamics of the neurons are described in §3. In §4.1, the dynamics within an SCG are modelled to generate a time series for the firings of each vertex within an SCG. In §4.2, we use the inter-spike intervals for a single neuron to construct a sequence of vectors according to the standard delay-coordinate embedding method [21]. Assembling these vectors as a matrix, we identify an upper bound for the embedding dimension *μ* for the time series. After establishing the neuron dynamics, and the method to find the embedding dimension for an SCG, in §5, we apply this method to many randomly generated SCGs, with varying number of vertices *n*. In §6, we consider the case where an SCG receives regular input with period *p*. Under this circumstance, we show that sometimes the activity within the SCG does not settle towards a corresponding periodic system. Lastly, in §7, we discuss our findings: that the embedding dimension increases sublinearly with the size of the SCG, implying that the neural network is more efficient when connected as demonstrated in figure 2.

## 2. Our neural network

Consider a very large population of interacting neurons, from some region of a human brain, that together form a directed graph of one-way synaptic connections, representing the fact that neuron A’s firing is an influence upon, or input to, an immediate downstream neuron B’s firing state. At the microscopic level of single connections, the dynamic response of B to A’s firing behaviour must involve some measurable delay: for the time taken for electrical excitable waves to be transmitted out from A’s soma, out along its axon, across a synapse, and then in along B’s dendrites to reach B’s soma, plus the time taken for B’s soma to respond electrochemically. These time delays may well be the key to building the rich behavioural *capacity* of such networks in exhibiting distinctive, alternative, modes of processing (as has been observed in complementary approaches using delay-continuum field theories [22]).

Now consider the macro-scale wiring structure of the directed graph corresponding to a very much larger collection of millions of such neurons. Within that network each neuron is represented by a unique vertex.

We will consider various directed graphs, denoted by *G*, on *n* vertices, *V* ={*v*_*i*_ | *i*=1,…,*n*}, having at most one edge between each pair of vertices and no loops (edges from a vertex to itself). We denote the edge from *v*_*i*_ to *v*_*j*_ by the ordered pair (*v*_*i*_,*v*_*j*_). If there is a connected walk from *v*_*i*_ to *v*_*j*_ then we will say that *v*_*j*_ is downstream from *v*_*i*_ and that *v*_*i*_ is upstream of *v*_*j*_. A *strongly connected* directed graph *G* is one where any vertex is connected to any other vertex by at least one walk (so that every vertex is both upstream and downstream of every other vertex). For any such graph *G*, we let *A* denote the *n*×*n* adjacency matrix, where *A*_*ij*_=1 if and only if the edge (*v*_*i*_,*v*_*j*_) is present and is zero otherwise. If *G* is strongly connected then *A* is *irreducible*. In that case we must have $\sum_{k=1}^{n-1}A^{k}>0$. Alternatively, one may check for strong connectivity with two applications of the depth-first search algorithm (DFS) [23]: pick an arbitrary vertex, *v* say, run DFS once starting at *v* and establish that all other vertices can be reached downstream from *v*. Then reverse all the edge directions and rerun DFS starting at *v*, thus checking that all other vertices are upstream of *v*.

Within a very large directed graph, we may be able to identify (via network analysis) many such strongly connected, or ‘irreducible’, sub-graphs, here called strongly connected (sub-)graphs, SCGs for short (figure 2). They exist at an intermediate, mesoscopic, scale in-between the pairwise microscopic interactions and the macroscopic whole network. We assume that SCGs are *maximal* in the sense that they are not a proper subset of any larger SCG. Then in any connected directed graph, a maximal SCG defines a three-way partition of the remaining vertices: those that are upstream of the SCG (there is a directed path from such vertices to at least one, and hence every vertex in the SCG); those that are downstream (there is a directed path from at least one, and hence every vertex in the SCG); and other vertices (e.g. downstream of upstream vertices, or upstream of downstream vertices) with no direct paths to or from the SCG. Therefore, an SCG receives input from a perturbation from an upstream SCG, and produces output by perturbing an upstream SCG. If we try to reduce an SCG by removing one or more of its vertices, by definition such vertices will be both upstream and downstream of the SCG, and we will no longer have a well-defined partition. Note that these definitions and ideas are related to the Markov blanket concept, that separates *active*-internal states and external states, explored for networks of neurons in Friston’s work [24].

We address a simple question: what *work*, in terms of processing inputs to producing outputs, might such an SCG, made of couples firing (dynamical) entities, actually do? To begin, we consider the dynamics of information propagation defined on SCGs.

## 3. Excitable-refractory-delay dynamics on strongly connected sub-graphs

In this paper, we discuss how the dynamical system within SCGs typically has multiple nonlinear resonant modes. The dynamical system is composed of coupled nonlinear dynamical unit ‘processors’ at each vertex (in this case called firing, excitable, refractory neurons) and incorporate transmission delays (resulting in delay-dynamical systems). Essentially, we should think of an SCG as a forced system when it is stimulated. If stimulated just once, and then left to its own devices, the nodes in the SCG will pass around excitation with suitable delays, possibly forever and ultimately in an a-periodic or periodic fashion; or it may cease firing and return to equilibrium. In the former case, the connectivity of the network may allow the cyclic propagation of excitation around closed paths (cycles). Hence the attracting set should be expected to be diffeomorphic to a *μ*-dimensional torus, denoted by $T^{\mu }$, for some suitable choice of dimension *μ*>1. This is simply the cartesian product on *μ* copies of the circle $S^{1}$, parametrized by a *k*-vector of 2*π*-periodic phase variables *ϕ*=(*ϕ*_1_,…,*ϕ*_*μ*_)^*T*^. We expect such an attractor will support a winding dynamic, where all phases continually increase (the simplest case being $\dot{\phi \;}\;=\omega$, some strictly positive winding rates vector in $R^{\mu }$). Indeed, we demonstrate directly that this is very often the case in estimating *μ* or rather an upper bound *m* for *μ*, from our detailed model calculations.

When such an SCG is forced with repeated, say *T*-periodic, stimulating pulses from some other upstream SCG then, for some such patterns of stimulation, it may produce a coherent, phase-locked mode of response. Such modes are firing patterns of behaviour distributed both in space (across the SCG) and in time. On the other hand, for some patterns of this stimulation the SCG may only respond incoherently (with no phase locking). This generic occurrence (or not) of phase entrainment behaviour would certainly be possible if the autonomous SCG system resulted in a single stable nonlinear limit cycle. In such a case, suitable periodic stimulations would result in phase locking on Arnold tongues, as described in Glass & Mackey [25]. However, the impact of the delays within the SCG is likely to increase the capacity of an SCG’s dynamics to exhibit alternative nonlinear cyclic modes, and have an attractor embedded in a torus, $T^{\mu }$, with *μ*>1. Recent work [26] considered the generalization of type-one and type-zero phase transition mappings (valid for instantaneous phase resetting in the limit cycle) to more diverse phase locking alternatives on the higher dimensional tori.

Generalizing for a moment, there are many applications of directed graphs where the vertices represent separate, similar, entities that are described in terms of *r* dynamical state variables, ${\text{x}}_{j}(t)\in R^{r}$ say, at vertex *v*_*j*_ (*j*=1,…*n*), with the graph’s edges describing the directed coupling between them and even where the dynamics feedback into the evolving edge couplings [27]. Typically, we will be in the situation where the state at vertex *v*_*j*_ is given by ${\text{x}}_{j}(t)\in R^{r}$ and depends only on the states of the vertices *v*_*i*_ that are immediately upstream of *v*_*j*_ (those *i* for which *A*_*ij*_=1). Such coupling may include time delays, *l*_*ij*_>0, corresponding to the time taken to receive input from *v*_*i*_ upstream. To be more specific, consider such a delay-differential equation having the form

$${\dot{\text{x}}}_{j}(t)=f({\text{x}}_{j}(t))+\sum_{i\;|\;A_{ij}=1}h({\text{x}}_{j}(t),{\text{x}}_{i}(t-l_{ij}))\;j=1,\ldots ,n.$$

Here, in the absence of coupling, $f:R^{m}\to R^{m}$ defines an autonomous nonlinear system at the *j*th vertex. The function $h:R^{m}\times R^{m}\to R^{m}$ describes the forcing from each of the immediate upstream vertices subject to the prescribed time lag.

Suppose we have such a system defined on a very large directed network, where some subset of the vertices defines a maximal SCG. Consider the dynamics on that SCG (ignoring any downstream activity which can have an influence on that of the SCG). Once ‘kick started’, and having no further input from any vertices upstream of the SCG, it will propagate activity (the separate finding of its various neurons) autonomously around itself. The necessary appearance of cycles (at least one) within such a strongly connected subnetwork means that quasi-periodic behaviours will dominate at large times as the maximal SCG continually passes activity around, stimulating and re-stimulating itself, over and over.

Returning to the specifically application we have in mind, this is a useful model for a neuronal network within part of the human brain, where the vertex dynamics represented by *f* are given by the Hodgkin–Huxley equations, the FitzHugh–Nagumo equations, or any other proposed excitable-refractory dynamical system [28]. Then the transmission time-lag represents the time taken for the electrical membrane depolarization waves to travel from one neuron centre (the soma) out along its axon, across a synapse and then inwards via the dendrite of a connected neuron, stimulating it to spike (or not) at its own soma. Of course, if activity runs in a cycle and returns to a neuron that has fired previously, it may arrive during the neuron’s refractory phase—during which it cannot be re-stimulated. If the cyclic propagation takes some time longer, the stimulus will return after the neuron has recovered and this can go again. When we perform numerical experiments solving the full delay differential equations above for Hodgkin–Huxley equations or FitzHugh–Nagumo dynamics this is exactly what is observed. Certain cycles may not be viable while others are, and the whole is a nonlinearly coupled system of such cycles that, once kick started, eventually settles into a long-term quasi-periodic attractor.

An extreme version of the above delay-dynamical system is to assert that *f* is such that:

(i) when perturbed sufficiently from its locally stable equilibrium state, it exhibits a very, very fast spike (that we may assume is instantaneous compared to the time scale of the transmission time-lags), followed by a fixed time-period, *δ*, during which the system cannot be re-stimulated (called a refractory period);
(ii) the coupling terms, represented by *h*, ensure that if any vertex immediately upstream should spike then, after the requisite time-lag, this is enough to excite the receiving vertex to spike immediately, providing that this stimulus does not arrive during a refractory period.

If we treat the spikes as instantaneous pulses, then it is enough to keep track of the firing times (and the consequent refractory periods) for each vertex. This is a far less expensive calculation than resolving the original delay differential equations with its wide multiplicity of time lags, and a division between the fast (pulse firing) and slow (neuron to near lagged preparations) time scales.

In this paper, we adopt this discrete approach to generate long-term behaviour (the inter-spike firing times for all vertices) for a very large variety of strongly coupled directed networks. All of the networks will be made of similar neuronal graphs, where we keep the expected vertex in and out degrees, *z*, to be constant, so that locally all networks appear similar regardless of overall size. The result of this work is to show that as the size, *n*, of such networks is increased (while *z* is fixed), the degrees of freedom exhibited by the long-term dynamical behaviour increase only sublinearly. The corollary to such a claim is that with limited volume and energy available, there is a diminishing marginal return on increasing the size of any strongly coupled subnetworks. Consequently, directed neuronal networks within human brains would have evolved so as to be wired with many, small maximal SCGs, rather than exhibiting any occasional large (or giant) ones. Large maximal SCGs simply do not provide a requirements increase in the variety of dynamic behaviour (degrees of freedom representing information and information processing within the macro-scale network) that would be worthy of the investment (of volume and energy, both of which are constrained).

In making such simulations, it becomes necessary to sample and simulate dynamics on a very large number of strongly connected networks as their size, *n*, is increased over orders of magnitude while *z* is held constant. This is a challenging topic in itself, as random networks of a given size are rarely strongly connected. We use a method of ‘edge swapping’ to generate Markov chains within the set of strongly connected directed graphs having given degrees distributions, so as to generate candidate SCGs for large *n*. This is set out in appendix A.

## 4. Methods

### (a) Discrete modelling

Consider a strongly connect directed graph representing connected neurons incorporating an excitable-refractory-delay dynamic. We will assume that a single firing pulse (measurable in membrane potential) is an instantaneous single spike event. Following each such spike, the neuron sits in a refractory state and cannot be re-stimulated for a time interval of length *δ*>0.

Once a neuron spikes that pulse is propagated out to its immediate downstream connections. These pulses arrive after a requisite transmission time lag. The time lags will be drawn independently and identically from a suitable distribution, and are assumed to be large compared with the pulse width, and this leads to our assumption that the pulse is an instantaneous spike. Upon arrival at each downstream neuron it is assumed that (i) if the downstream neuron is not sitting within a refractory period then the incoming spike is strong enough to cause the downstream neuron to spike itself and (ii) if the downstream neuron is sitting within a refractory period then the incoming spike has no effect.

So, given the network (the adjacency matrix) and the corresponding matrix of time lags, for each directed edge, we may iteratively update the whole network’s spike times by the following dynamical model.

At any time *t*=*t*_1_, for each neuron, we will have a list of all of the historical spike times that have already occurred (in the past, for *t*<*t*_1_), and also a list of possible spike times (in the future, for *t*>*t*_1_) when each neuron will receive an incoming spike (propagated from an upstream spike in the past). We select the next earliest future incoming spike times for any of the neurons, at some time $t_{2}^{\left(a\right)}>t_{1}$ say, and then we check whether that corresponding receiving neuron will be in its refractory period at that $t_{2}^{\left(a\right)}$ (due its own most recent earlier spike in its historical list). If it is, then that receiving neuron will not spike, and we do not record a firing at time $t_{2}^{\left(a\right)}$. Instead, we select the second earliest future incoming spike times for any of the neurons, at some time $t_{2}^{\left(b\right)}>t_{1}$. This process is repeated until the receiving neuron is not in a refractory period for some time $t_{2}^{\left(\cdot \right)}$. Then we assume that it spikes at $t_{2}^{\left(\cdot \right)}=t_{2}$, we move the clock on to *t*=*t*_2_, we update the receiving neuron’s corresponding list of historical spike times to include the spike at *t*_2_, and we generate some new possible future spike times for each of its immediately downstream neurons at times given by *t*_2_ plus the relevant transmission lag, and add these to the full list of possible future spike times. Then we iterate.

To start up, we pick a single neuron and we assume (i) it spikes at *t*=0, so it has a single element in its list of future spike times, (ii) that all neurons are not refractory at *t*=0 (in effect they have no historical firing events, and thus have not fired recently within time *δ*) and (iii) that all of the neuron’s lists of future possible firing times are empty.

**Figure 3.**
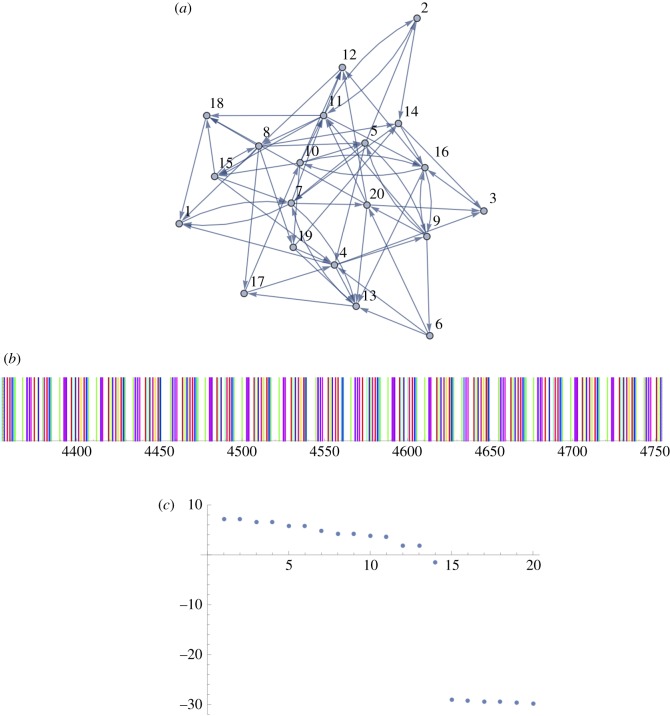
Here the number of vertices in the SCG is *n*=20, the average in/out degree is *z*=3, and the refractory period is *δ*=20. The diameter of the graph is 6. Transmission lags are identically and independently distributed (i.i.d) uniformly in [50,100]. (*a*) The strongly connected graph. (*b*) The 20 neural spike trains times (after suitable a burn-in period) with spikes at each vertex coloured separately—not obviously periodic. (*c*) The natural log of the first 20 eigenvalues of the lag-correlation matrix (with windows of dimension *k*=80) for the inter-spike intervals (observed at a single vertex), indicating that the attractor is embeddable in 14 dimensions (the mean inter-spike interval is 22.08 at all vertices).

In figure 3, we show a simple example. Following a single kick-start spike at *t*=0 at vertex 1, we depict the resulting firing times as vertical bars, coloured by vertex: essentially 20 neural spike trains (figure 1) differentiated by colour, not location on the *y*-axis. The result is quasi-periodic.

### (b) Embedding analysis

In order to analyse such results, we consider the sequence of interspike intervals q (at a single vertex) so as to estimate a suitable embedding dimension for the state space reconstruction of the long-term dynamical behaviour, close to the attractor. This is based on generalizations for the Takens embedding theorem [21] and analogous ideas [29], and is neatly summarized in Bradley & Kantz [30]: singular spectrum analysis enables such a reconstruction. The standard method is based on delay-coordinate embedding, where a series of ‘windows’, each containing *k* successive past values of a scalar observable (from a dynamical system), forming a vector in $R^{k}$. Such delay-coordinate embedding usually requires the data to be evenly sampled in time. However, when the data consist of discrete events, such as the spikes here, one can justify the application of window-embedding to the sequence of interspike intervals itself [31,32]. Therefore, the corresponding lag-correlation matrix

$$\left(\begin{array}{cccc} q_{1} & q_{2} & \ldots & q_{k}\\ q_{2} & q_{3} & \ldots & q_{k+1}\\ q_{3} & q_{4} & \ldots & q_{k+2} \end{array}\right)$$

is formally missing not only an embedding dimension *m* (because that is what we seek), but also a time delay parameter. Now, the *k* eigenvectors of the lag-correlation matrix define empirical orthogonal dimensions, while the corresponding eigenvalues (all positive and real by definition) account for the partial variance of the embedded point cloud in the corresponding direction. Signal-to-noise separation can be obtained by simply locating a significant break in the ordered list of eigenvalues (pink or white noise would produce a natural decay or plateau of the spectrum, without such large breaks). The position *m* (<*k*) after which this spectral break occurs is thus an estimated upper bound for the actual dimension *μ* of the underlying deterministic dynamics, and the corresponding first *m* eigenvectors define a set of coordinates with which to project and embed the sequence of windows. Thus, *m* represents an estimate of the dimension of the embedding space containing the underlying attractor.

Of course if the attractor has actual dimension equal to *μ* then typically one may require a trial window-embedding dimension of at least *k*>*m*, which could be as large as 2*μ*. However, we might hope that *m* is closer to *μ* in our calculations, since $T^{\mu }$ is certainly embeddable in $R^{\mu +1}$.

Consider the example given in figure 3. The series of firing times, the spike train, for the SCG are shown coloured by vertex. We take the window length to be *k*=80. Considering the ordered list of the (logarithms of the) eigenvalues of the lag-correlation matrix, there is an obvious break after *m*=14. This leads us to assert that *μ*≤14 and is very likely to be 13, corresponding to an attractor in the form of $T^{13}$.

Hence for any sampled SCG, generated on *n* vertices, we will generate an appropriate set of transmission lags, independent and identically, from a given distribution and calculate firing times following a kick start. Then we will employ the above state space embedding methodology to estimate an upper bound, *m*, for the actual dimension, *μ*, of the corresponding attractor. In the next section, we consider how *m* behaves as the number of vertices, *n*, varies, while keeping *z*, the expected in and out degree of all vertices, constant.

## 5. A large-scale survey

In this section, we carry out a large survey, analysing SCGs, with excitable-refractory delay dynamics, across a range of sizes having similar edges densities (as measured by the mean in and out vertex degree, *z*), a fixed value for the refractory period, *δ*, and similar transmission lags. For each SCG dynamic, we estimate *m* and thus we sample the conditional distribution *P*(*m* | *n*,*X*), where *X* stands for everything that we prescribed for the SCGs and the associate neuronal dynamics. We will examine how this distribution varies as the number of vertices, *n*, varies over orders of magnitude.

More precisely, we assume that (i) the refractory period *δ*=30, (ii) all transmission lags are identically and independently distributed (i.i.d.) uniformly [50,100] and (iii) the expected in and out degree of all vertices, *z*, is approximately equal to 3.

When *n* is large it is not trivial to sample from the corresponding set of strongly connected, directed, graphs with mean in and out degree equal to 3. Consequently, we have adopted a method based on edge swapping that enables the generation a Markov chain of such irreducible graphs, starting out at highly contrived graphs and evolving towards more homogeneous graphs, with the graph diameter decreasing along the chain. This allows the generation of networks with relatively small diameters over many hundreds of vertices and is set out in appendix A.

**Figure 4.**
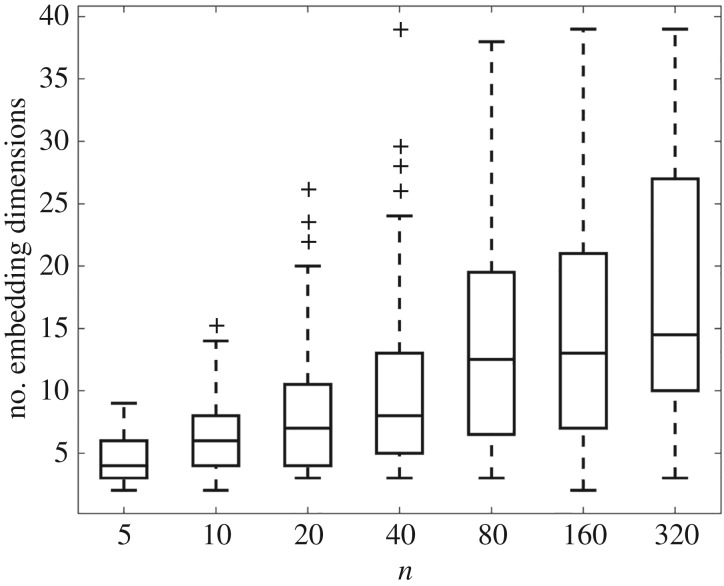
The median (middle line), interquartile range (box edges) and range (whisker edges) of the observed conditional distribution for *m*, *P*(*m* | *n*,*X*), on the vertical axis, as the number of vertices, *n*, varies along the *x*-axis. For each value of *n* (the number of vertices in the SCG), we sampled 100 SCGs and used refractory period *δ*=30, and average in/out degree *z*=3, with all transmission lags i.i.d uniformly in [50,100]; and *k*=80. The number of embedding dimensions for the SCGs scales sublinearly with *n*.

Some typical results are shown in figure 4. It is clear that *m* increases sublinearly with *n*. This is an important insight because below we argue that *m* gives a measure of the different types of behaviour that a dynamic SCG can exhibit. If the dynamic on the SCG is forced at different frequencies, and at different vertices internally, then it may respond in different ways. If *m*=2, the attractor being a limit cycle, with *μ*=1, then the dynamics either entrain with the periodic forcing or do not. The situation for higher dimensional tori is somewhat more complicated [26]—nevertheless, there are likely to be different modes that can be resonant with distinct periodic forcing. Those resonant modes of response may also depend on the particular internal vertex at which the forcing from some upstream vertex (within some upstream SCG) is received.

## 6. Resonant modes of strongly connected sub-graphs

We consider an SCG with an excitable-refractory-delay dynamic defined on it, as in previous sections. Rather than kick starting it once and then allowing it to run to a dynamic equilibrium, here we force it periodically with time period *p*>0. For each period, we estimate whether the resulting behaviour of the dynamic responds coherently, and becomes entrained, exhibiting periodic behaviour itself with period equal to an integer multiple of *p*. If it does not do so then the dynamic may become chaotic and thus remains relatively incoherent.

Consider the system presented in figure 3. We force it periodically by stimulating a spike every *p* units of time arriving at vertex 1 (as if arriving from some upstream vertex, within an upstream SCG, that is firing with period *p*). If vertex 1 happens to be within a refractory period when it is stimulated then that forcing pulse has no effect. For different values of *p*, we can observe whether the system becomes periodically entrained, that is, when the system’s exhibited period to forcing period ratio is of the form *K*:1 (and so the system response has period *Kp*).

**Figure 5.**
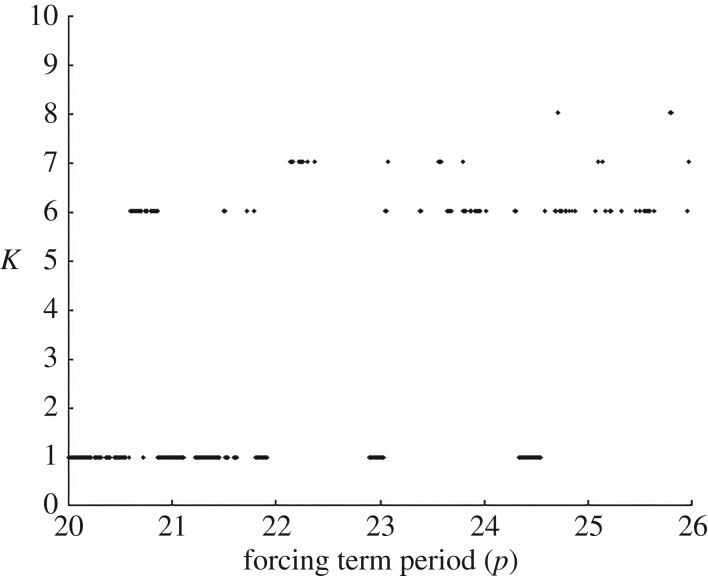
When the SCG is periodically stimulated with period *p* (the *x*-axis), the SCG dynamic generally settles to a period of length *Kp*. Note there are ‘gaps’ where no entrainment is observed, while between those gaps the entrained system’s period to forcing period ratio is 1 : 1 (with period *p*), implying ‘coherence’. When the ratio is not 1 : 1, a longer sequence of firings may be required, or the system is chaotic.

In figure 5, we depict the situation for a wide range values of *p*. In general, after 100 000 firings, the SCG dynamical system appears to respond ‘coherently’ and becomes entrained for certain intervals of the forcing period, *p*. The ‘gaps’ between the intervals imply either a higher order of entrainment or a chaotic system, and so it does not become entrained.

## 7. Discussion

In this paper, we considered a directed graph model for the human brain’s neural information processing architecture that is based on a collection of small, directed SCGs. We assumed that there are transmission delays in neuron-to-neuron stimulation and found that these are critical in increasing the capacity of the behaviour that each SCG can support. We demonstrated that, in isolation, the SCGs made of delay-coupled neuron dynamics typically have attractors that are equivalent to continual winding maps over relatively low-dimensional tori.

We carried out a large-scale survey of such SCGs, containing *n* neurons, where *n* varies over orders of magnitude, while the expected vertex in and out degrees are fixed (so that all of the networks, when considered locally, have the same density of connections). For each such delay-differential system, the dimension *μ* of the corresponding attractor for the dynamical system represents a limit on the range of distinct modes of behaviour that can be exhibited. We found that the conditional distribution for the embedding dimension, *m*, for the attractor of dimension *μ*≤*m*, scales sublinearly with *n*. Thus, there may be benefits in brains having evolved so as to have a larger number of *m*-small irreducible sub-graphs, rather than fewer *n*-large irreducible sub-graphs or giant components.

We conjecture that this result is a function of the architecture and is not particularly sensitive to (i) the particular choice of excitable-refractory dynamics employed, provided it involves relatively fast spikes and relatively slow neuron-to-neuron transmissions or (ii) the methodology employed to estimate *m*, an upper bound for the dimension *μ* of the long-run attractor. Indeed in future work we will demonstrate an identical result with continuous time delay differential equations and an examination of the Fourier spectra of the consequent long-term firing patterns.

When the SCGs are coupled up at the macroscopic scale, where each is driven by a few other SCGs immediately upstream, we discussed the generalization of phase response curves and the existence of multiple distinct resonances. The point is that SCGs can only become phase locked and entrained with their drivers for certain resonant ranges of periodic stimulation. Thus, each SCG behaves as a kind of analogue filter, and thus an amplifier and propagator of a distinct and discrete number of alternative time-dependent behaviours. The nonlinearity of the dynamics means that resonant modes might be like competing hypotheses (in a Bayesian setting) with one winning out as more and more forcing stimulus is applied.

## Figures and Tables

**Figure 6.**
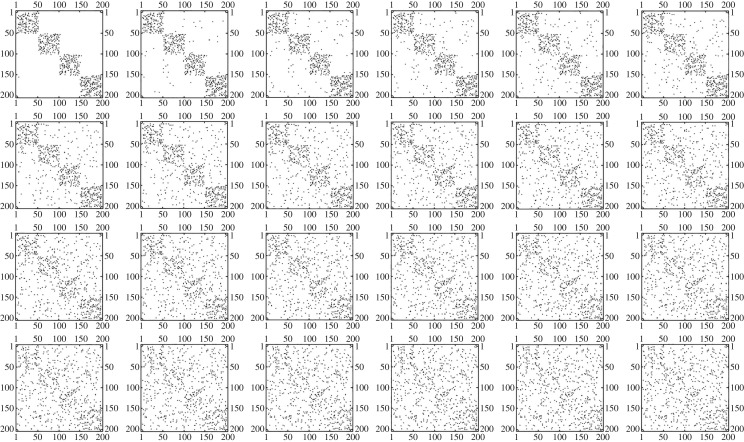
A random walk through $H_{n}$ with *n*=200 vertices, starting from a graph with *K*=4 separate irreducible clusters, each with *R*=50 vertices and *z*=3, and joined together by a single directed edge cycle. We show the adjacency matrices for the graph after 20 successive edge swaps (applications of *F*), demonstrating that the method ensures average in/out degree of *z*=3 while homogenizing spread of edges.

**Figure 7.**
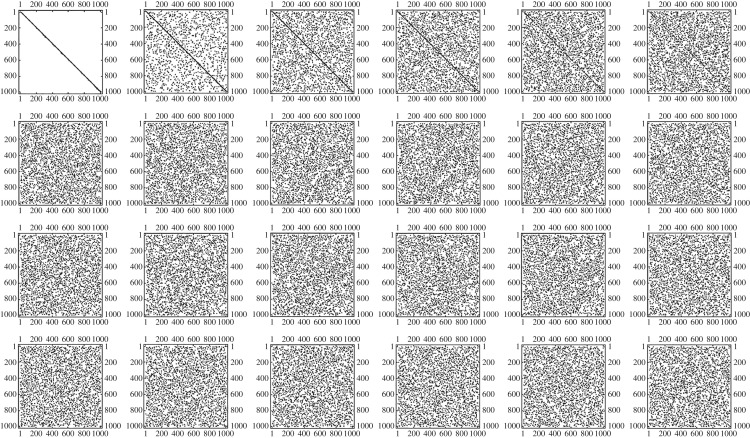
A random walk through $H_{n}$ with *n*=1000, starting from a range-dependent random graph [39] where edge (*v*_*i*_,*v*_*j*_) (*i*≠*j*) is present with probability $p_{ij}={\left(\frac{1}{3}\right)}^{\left(|i-j|-1\right)}$ so both in and out degrees have an expected value of *z*=3 (there are actually 3018 edges). There are 1000 edge swaps (successive applications of *F*) between each successive graph. We show the adjacency matrices for successive graphs.

## Data accessibility

There are no datasets supporting this article.

## Appendix A. Generating strongly connected sub-graphs for large *n* and fixed *z*

Let $G_{n}$ denote the set of all directed graphs over *n* vertices *V* ={*v*_*i*_ | *i*=1,…,*n*}. Let $H_{n}\subset G_{n}$ be the subset of strongly connected directed graphs on *V* . A *random directed graph* is a probability distribution defined over $G_{n}$. The distribution for certain classes of random graph are easy to describe, and thus easy to sample from. For example, if each edge (*v*_*i*_,*v*_*j*_) is present independently with known probability *p*_*ij*_ then sampling from such a distribution is straightforward. If the *p*_*ij*_ are the same for all possible edges, say *p* (except being zero when *i*=*j*), then we have the homogeneous random directed graph that is usually denoted by *D*(*n*,*p*).

In this paper, we wish to sample elements from $H_{n}$. Suppose we set *p*_*ij*_=*p*(*n*), some constant probability, for *i*≠*j* (zero otherwise), then we might generate a graph from *D*(*n*,*p*(*n*)) over $G_{n}$ and then test it for irreducibility. Further, we may take *p*(*n*)=*z*/(*n*−1) so that the expected in degree and out degree for each vertex remains the same, equal to *z*, as we vary *n*.

For *n* relatively small this ‘trial-and-error’ procedure will be fine, yet it quickly becomes inefficient for *n* large (in fact, greater than 100, say). To see this, suppose we generate such a random directed graph on *n* vertices. Then the probability that a given vertex has zero in degree (or equivalently has zero out degree), implying that the whole is not strongly connected, is *q*=(1−*z*/(*n*−1))^*n*−1^. As n→∞ we have q→exp⁡(−z). Hence the probability that all vertices have both in and out degree greater than or equal to one is approximately $(1-\exp{\left(-z)\right)}^{2n}.$ This last is an upper bound on the probability that the graph is strongly connected, and shows why the ‘trial-and-error’ approach is doomed for large *n*.

We follow the method discussed by Viger & Latapy [33,34] and set within a wider frame by Blitzstein & Diaconis [35]. This in turn is based on the idea of edge switches (or swapping): taking two (or more) edges and swapping their destination vertices around to create replacement edges (see [36] and the discussion and references therein). This idea is highly traditional within graph theory, as switching preserves both the in and out degrees of all vertices and hence is compatible with the configuration model. Such as strategy, originally proposed by Miklos & Podani [37], was developed by Tabouriera *et al.* [36] and Artzy-Randrup & Stone [38] to other types of constrained subsets of directed graphs.

It is usually trivial to create directly an element of $H_{n}$ with a given in and out degree distribution. But these will inevitably be highly contrived, since we would need to ensure the existence of cycles, or possibly a Hamiltonian cycle. So we will start off with some such artificially structured element and then successively transform it into something more homogeneous, and less contrived, having a smaller diameter, say.

Suppose we have a graph $G\in H_{n}$. Then we may modify *G* randomly to create a new graph $G^{\prime }\in H_{n}$ according to the ‘switch and test’ algorithm below. We shall write *G*′=*F*(*G*) where *G*′ is drawn from a conditional probability distribution defined over $H_{n}$, say *P*(*G*′ | *G*). We shall select edge switches randomly and then test whether the new proposed graph maintains strong connectivity.

*Step 1*. Select two edges randomly, with equal probability, from the set of edges in *G* , say (*v*_*a*_,*v*_*b*_) and (*v*_*α*_,*v*_*β*_).
*Step 2*. Propose new edges (*v*_*a*_,*v*_*β*_) and (*v*_*α*_,*v*_*b*_) (swapping the end vertices over).
*Step 3*. If either (*v*_*a*_,*v*_*β*_) or (*v*_*α*_,*v*_*b*_) is already in *G*, or is a loop, go back to Step 1.
*Step 4*. Form the graph *G*′ from *G* by deleting the old edges (*v*_*a*_,*v*_*b*_) and (*v*_*α*_,*v*_*β*_) and inserting the new edges (*v*_*a*_,*v*_*β*_) and (*v*_*α*_,*v*_*b*_).
*Step 5*. Check whether *G*′ is strongly connected. If it is not then go to Step 1. If it is then EXIT.

The test in Step 5 can be to simply check the strict positivity of one of the matrix functions of the adjacent matrix *A*′, for *G*′, as discussed above. But in fact we can do better, since we only need to check that there are walks from *v*_*a*_ to *v*_*b*_ and from *v*_*α*_ to *v*_*β*_ in *G*′, since any walk in *G* connecting any pair of vertices that previous relied on either or both of the deleted edges will be able to take these alternatives in *G*′: hence *G*′ will be strongly connected. This can be achieved by two applications of the DFS algorithm starting from *v*_*a*_ and *v*_*α*_, respectively [23].

Then applying *F* successively we create a Markov chain of elements in $H_{n}$. If *G* contains *m* edges then so does *F*(*G*); similarly, it is clear that the in and out degrees of all vertices is unchanged by *F*. Hence if we require a strongly connected directed graph with any given distribution of in and out degrees, we first generate one by hand, and then apply *F* as often as we wish.

For example, we have observed that it is easy, by trial and error, to create strongly connected directed graphs on only 50 vertices, with *z*=3 (yet it is very hard to do so with 200 hundred vertices). So we may proceed as follows. First we generate *K*>2 strongly connected graphs each on only *R* (≪100) vertices, with a fixed expected in and out degree, *z* say. Then we form-up a single graph from their disjoint union with *n*=*K*.*R* vertices in total. We call the separate subgraphs ‘clusters’. Next we add *K* extra edges connecting up the clusters in a directed cycle as follows. We order the clusters into a cycle, and then create a new directed edge by selecting a random starting vertex from one cluster and a random ending vertex from the next cluster in the cluster cycle. The resulting graph *G* is trivially in $H_{n}$, as required.

Next we may apply *F* many times over. An example of this process is shown in figure 6 in the case that *R*=50, *K*=4, *z*=3 and thus *n*=200; and we depict the corresponding adjacency matrix after each 20 successive edge swaps (applications of *F*). Each graph has 602 edges. After 480 swaps, we arrive at a graph (bottom right) where the clusters’ ‘block’ structure has become finely dispersed.

In figure 7, we give an example starting out from a simple range-dependent random graph [39], where edge (*v*_*i*_,*v*_*j*_) (*i*≠*j*) is present with probability $p_{ij}={\left(\frac{1}{3}\right)}^{\left(|i-j|-1\right)}$ (so that neighbours are always directly connected but second neighbours are directly connected with probability $\frac{1}{3}$, etc.). The expected in and out degree, *z*, is thus 3. The strongly connected graphs in the Markov chain all have 3018 edges.

The Markov chain ranges over the subset of graphs in $H_{n}$ having the exact joint distribution of in and out degrees specified for the starting graph. Suppose we focus on a single directed edge, *E*_0_, in the starting graph, out of *m* (larges) given edges in total. Then after *km* switches (*k*>1), the probability that *E*_0_ is unaffected is approximately (1−2/*m*)^*mk*^→*e*^−2*k*^. So if k>(ln⁡m)/2, i.e. the total number of switches is more than (mln⁡m)/2, then we expect that less than one of the original edges will still remain. For *m*=3000, we should take *k*=5 and perform 15 000 swaps. In figure 7, this corresponds to the 15th adjacency matrix shown.

In each of the above examples, the average degree (in and out) is approximately *z*=3. So the diameter, *d*, of each of the networks appearing within those Markov chains is bounded below by that, *d*_0_ say, for a tree (with no cycles and each vertex having *z* edges out). We will have $d_{0}>-1+\log_{z}(z-1)n$, which is around 6 for the parameters used in defining the chain of networks shown in figure 7. In fact in those networks, the diameter starts out very high and reduces down to 10 after around 2000 swaps (the third adjacency matrix shown) and remains there.
